# Iodine nutrition status and its association with microvascular complications in urban dwellers with type 2 diabetes

**DOI:** 10.1186/s12986-020-00493-5

**Published:** 2020-08-17

**Authors:** Chi Chen, Yi Chen, Hualing Zhai, Fangzhen Xia, Bing Han, Wen Zhang, Yuying Wang, Heng Wan, Ningjian Wang, Yingli Lu

**Affiliations:** Institute and Department of Endocrinology and Metabolism, Shanghai Ninth People’s Hospital, Shanghai JiaoTong University School of Medicine, Shanghai, 200011 China

**Keywords:** Iodized salt, Type 2 diabetes, Diabetic kidney disease, Urinary iodine concentration, Epidemiology

## Abstract

**Background:**

The principal function of iodine acts on thyroid function, but in recent years, the role of iodine deficiency in metabolism has also been gradually revealed. We aimed to investigate the current status of iodized salt consumption and urinary iodine concentration (UIC) in an urban Chinese population with type 2 diabetes, and to further explore whether UIC was associated with diabetic microvascular complications.

**Methods:**

Four thousand five hundred fifty-nine subjects with diabetes from 7 communities in downtown Shanghai were enrolled in the cross-sectional Metal Study in 2018. UIC was detected using an inductively coupled plasma-mass spectrometer. Diabetic kidney disease (DKD) was defined as urinary albumin-to-creatinine ratio (UACR) > 30 mg/g or estimated glomerular filtration rate < 60 mL/min/1.73 m^2^. Diabetic retinopathy (DR) was evaluated by high-quality fundus photographs and was remotely read by ophthalmologist.

**Results:**

The median UIC of subjects with diabetes was 115.4 μg/L (78.9–170.8) in downtown Shanghai. Among all the subjects, 52.7% consumed non-iodized salt and 40.4% were iodine deficient. Iodine deficiency (UIC < 100 μg/L) was associated with an increased odds of DKD (OR 1.17; 95%CI 1.01–1.37) after adjustment for age, sex, education, current smokers, BMI, HbA1c, duration of diabetes, dyslipidemia, thyroid-stimulating hormone and free thyroxine. No association was observed between UIC and DR after multivariable adjustment.

**Conclusions:**

A concerning number of subjects with diabetes consumed non-iodized salt and suffered from iodine deficiency in coastal regions of China. Low UIC might be a risk factor for DKD, which should be further confirmed by longitudinal prospective studies.

## Introduction

Type 2 Diabetes Mellitus (T2DM) has become a serious global health care burden, causing microvascular complications which are associated with increased disability, reduced quality of life and life expectancy [1, 2]. It was estimated that there were 451 million cases of adult diabetes worldwide in 2017 and the number was projected to increase to 693 million by 2045 [3], leading to high incidence and prevalence of microvascular complications. Approximately one third with T2DM will develop diabetic retinopathy (DR), and one quarter will develop diabetic kidney disease (DKD) [4]. Thus, it is critical to identify and control some novel modifiable risk factors contributing to microvascular complications in patients with T2DM.

Iodine is an indispensible micronutrient for the synthesis of thyroid hormones. Iodine deficiency (ID) in early life impairs neurodevelopment, and also has many adverse effects throughout various life stages [5]. Over the past 20 years, substantial progress has been achieved in the worldwide effort to eliminate iodine deficiency disorders (IDD) by salt iodization program. However, this program is vulnerable and requires a long-term commitment from governments. In several countries where ID had been once eliminated, salt iodization programs were discontinued and ID has now reappeared [6].

The principal function of iodine acts on thyroid function, but in recent years, the role of ID in metabolism has also been gradually revealed. Analysis of data from the National Health and Nutrition Survey (NHANES) 2007–2012 found that in US adults, low urinary iodine concentration (UIC) was associated with dyslipidemia [7] and coronary artery disease [8]. Moreover, O. S. Al-Attas et al. [9] reported that UIC is markedly decreased in T2DM and urinary iodine was negatively associated with insulin resistance in patients with T2DM. Most recently, Mingyue Jin et al. [10] also found that at a lower UIC (< 100 μg/L), the prevalence of diabetes significantly increased relative to an UIC of 100–299 μg/L. Animal studies also showed that iodine supplementation could reduce the blood glucose levels and improve the insulin sensitivity in goats [11]. However, the role of iodine nutrition on diabetic microvascular complications has not been studied.

The aim of this study was to investigate the current status of iodized salt consumption and UICs in an urban Chinese population with T2DM, and additionally whether UIC is associated with diabetic microvascular complications including DKD and DR.

## Methods

### Study population

The cross-sectional METAL study (Environ**me**ntal Pollu**ta**nt Exposure and Metabo**l**ic Diseases in Shanghai, www.chictr.org.cn, ChiCTR1800017573) was launched to investigate the association between iodine nutrition and microvascular complications in Chinese adults with diabetes. We recruited study participants from seven communities in Huangpu and Pudong new district, Shanghai, China. Huangpu district, located in downtown Shanghai, is the administrative, economic and cultural center of the metropolitan coastal city [12]. Pudong new district is the symbol of China’s reform and opening-up [13]. We randomly selected half of patients with diabetes from the registration platform in each community healthcare center. Chinese citizens ≥18 years old who had lived in their current area for ≥6 months were included. In August 2018, a total of 4813 subjects with T2DM who were 23–99 years of age received an examination. Participants with missing UIC values (*n* = 254) were excluded. Finally, 4559 participants were involved in the present analysis.

The study received ethical approval from the Ethics Committee of Shanghai Ninth People’s Hospital, Shanghai Jiao Tong University School of Medicine. All procedures followed were abided by the ethical guidelines of the 1975 Declaration of Helsinki as reflected in a priori approval by the appropriate institutional review committee. Informed consent was received from all participants included in the study prior to the data collection.

### Measurements

The same well-trained and experienced personnel in SPECT-China study [14–16] used a questionnaire to collect information on socio-demographic characteristics, education, medical history, family history, and lifestyle risk factors. Weight and height were measured using a balance beam and a vertical ruler in light clothing and without shoes. Body mass index (BMI) was calculated as the ratio of weight in kilograms divided by height in meters squared. Current smoking was defined as having smoked at least 100 cigarettes in one’s lifetime and currently smoking cigarettes [17]. Especially, “For the past three years, which type of salt was used in your family” was applied to collect information about type of salt. Three options for this item were provided: (1) only iodized salt; (2) only non-iodized salt; (3) both.

Blood samples were drawn between 6:00 am and 9:00 am after an overnight fast. Blood was refrigerated immediately after phlebotomy, and in 2 hours it was centrifugated and the serum was aliquoted and frozen in a central laboratory. Glycated hemoglobin (HbA1c) was measured by high-performance liquid chromatography (MQ-2000PT, Medconn, Shanghai, China). Fasting plasma glucose, serum creatinine, triglycerides, total cholesterol, high (HDL) and low-density lipoprotein (LDL) were performed with a Beckman Coulter AU 680 (Brea, USA). Serum thyroid-stimulating hormone (TSH) and free thyroxine (FT4) were measured by electrochemiluminescence (Roche, E601, Germany).

Morning fasting spot urine samples collected were refrigerated immediately and frozen at − 20 °C in 2 hours. UIC was detected using an inductively coupled plasma-mass spectrometer (ICP-MS, No. 7700x, Agilent Technologies Inc., USA). The concentrations of urine albumin and creatinine were determined with a Beckman Coulter AU 680 (Brea, USA) using a turbidimetric immunoassay and an enzymatic method, respectively. Urinary albumin-to-creatinine ratio (UACR) was calculated as the urinary albumin concentrations divided by the urinary creatinine concentrations and expressed in mg/g.

DR screening was evaluated by mydriatic binocular indirect ophthalmoscopy (Topcon TRC-NW400 Non-Mydriatic Retinal Camera, Oakland, USA). Fundus photographs were read by an experienced ophthalmologist specialized in retina.

### Outcome definition

Dyslipidemia was defined as total cholesterol ≥6.22 mmol/L (240 mg/dL), triglycerides ≥2.26 mmol/L (200 mg/dL), LDL ≥ 4.14 mmol/L (160 mg/dL), HDL < 1.04 mmol/L (40 mg/dL), or self-reported previous diagnosis of hyperlipidemia by physicians, according to the modified National Cholesterol Education Program-Adult Treatment Panel III.

The estimated glomerular filtration rate (eGFR) was calculated using the Chronic Kidney Disease Epidemiology Collaboration (CKD-EPI) equation for “Asian origin” [18]. As suggested by American Diabetes Association (ADA), high UACR was defined as UACR ≥30 mg/g, reduced eGFR as eGFR < 60 ml/min/1.73m^2^, and DKD as UACR > 30 mg/g or eGFR < 60 mL/min/1.73 m^2^ [19].

The internationally accepted DR classification by the “Global Diabetic Retinopathy Project Group” in 2003 was applied [20]. The classification was: no retinopathy, non-proliferative DR (intraretinal micro-aneurysms, hemorrhages, venous beading, prominent microvascular abnormalities) and proliferative DR (neovascularization or vitreous/preretinal hemorrhages).

### Statistical analysis

Statistical analysis was run with SPSS 24.0 (IBM Corporation, Armonk, NY, USA). General characteristics are presented as median with the interquartile range (IQR) for continuous variables or as proportion for categorical variables. Mann-Whitney U test and the Kruskal-Wallis test were used for comparison of two or more groups of non-normally distributed data. Pearson’s χ2 tests were performed to compare categorical variables.

The associations of UIC with elevation of UACR, reduction of eGFR, DKD and DR were analyzed with logistic regression analyses. The results are presented as odds ratio (OR) and 95% confidence intervals (CIs). Model 1 was unadjusted. Model 2 was adjusted for age, sex, education, current smokers, BMI, HbA1C, duration of diabetes, dyslipidemia, TSH and FT4.

## Results

### General characteristics of the study population

The general characteristics are presented in Table 1. The mean age of the study population was 67.0, nearly one half (53.9%) were women. About 45.5% of the study population were overweight or obese (BMI ≥25 kg/m^2^), and 17.9% were current smokers. The percentage of an educational level beyond high school was 50.9% and the average duration of diabetes was 9 years.

**Table 1:** General characteristics of study participants by UIC categories

Urinary iodine	Adequate	Low	More than adequate	Excessive	*P*
Age, yr	67 (61–72)	68 (63–73)	66 (61–71)	66 (60–71)	< 0.01
Women, %	50.1	57.9	55.2	52.3	< 0.01
UIC, μg/L	136.9 (118.1–164.4)	72.3 (57.3–85.2)	234.8 (214.9–262.1)	392.8 (335.0–604.6)	< 0.01
FPG, mmol/L	7.2 (6.1–8.6)	7.2 (6.2–8.5)	7.4 (6.4–9.0)	7.1 (6.1–8.5)	0.08
HbA1c, %	7.2 (6.5–8.1)	7.1 (6.5–8.1)	7.3 (6.6–8.2)	7.1 (6.5–8.0)	0.06
BMI, kg/m^2^	24.6 (22.6–27.1)	24.4 (22.4–26.9)	25.1 (22.8–27.8)	25.2 (23.0–27.5)	< 0.01
Duration of diabetes, yr	8 (3–15)	10 (4–16)	7 (3–15)	9 (3–15)	< 0.01
Current smokers, %	19.4	15.7	19.0	20.4	0.01
Beyond high school education, %	51.0	50.8	51.4	50.5	0.994
TSH, mIU/L	2.54 (1.75–3.56)	2.61 (1.84–3.73)	2.36 (1.65–3.45)	2.62 (1.74–3.74)	0.015
FT4, pmol/L	16.57 (15.25–18.13)	16.71 (15.25–18.21)	16.75 (15.26–18.48)	16.36 (15.02–17.92)	0.129
**Blood lipids**					
Total cholesterol, mmol/L	5.07 (4.29–5.85)	5.03 (4.26–5.86)	5.19 (4.36–5.93)	4.98 (4.12–5.82)	0.22
LDL-C, mmol/L	3.15 (2.55–3.72)	3.10 (2.54–3.69)	3.23 (2.62–3.76)	3.04 (2.50–3.61)	0.12
HDL-C, mmol/L	1.16 (0.98–1.36)	1.18 (1.02–1.39)	1.18 (1.02–1.38)	1.15 (0.98–1.34)	< 0.01
Triglycerides, mmol/L	1.51 (1.08–2.17)	1.53 (1.10–2.19)	1.58 (1.15–2.17)	1.58 (1.08–2.36)	0.33

Data are summarized as median (interquartile range) for continuous variables or as number with proportion for categorical variables

*UIC* Urinary iodine concentration, *FPG* Fasting plasma glucose, *HbA1c* Glycated hemoglobin, *BMI* Body mass index, *LDL-C* Low density lipoprotein cholesterol, *HDL-C* High density lipoprotein cholesterol, *TSH* Thyroid-stimulating hormone, *FT4* Free thyroxine

Urinary iodine concentrations: low, < 100 μg/L; adequate, 100 to < 200 μg/L; more than adequate, 200 to < 300 μg/L; excessive, ≥300 μg/L

Compared to those with adequate iodine nutrition, subjects with ID were slightly older and were more likely to be women. These subjects also had higher TSH, lower BMI and a lower percentage of current smokers. In addition, subjects with more than adequate and excess iodine nutrition were slightly younger and had higher BMI, but comparable TSH.

### Urinary iodine concentration and type of salt intake in the population

**Fig. 1:**
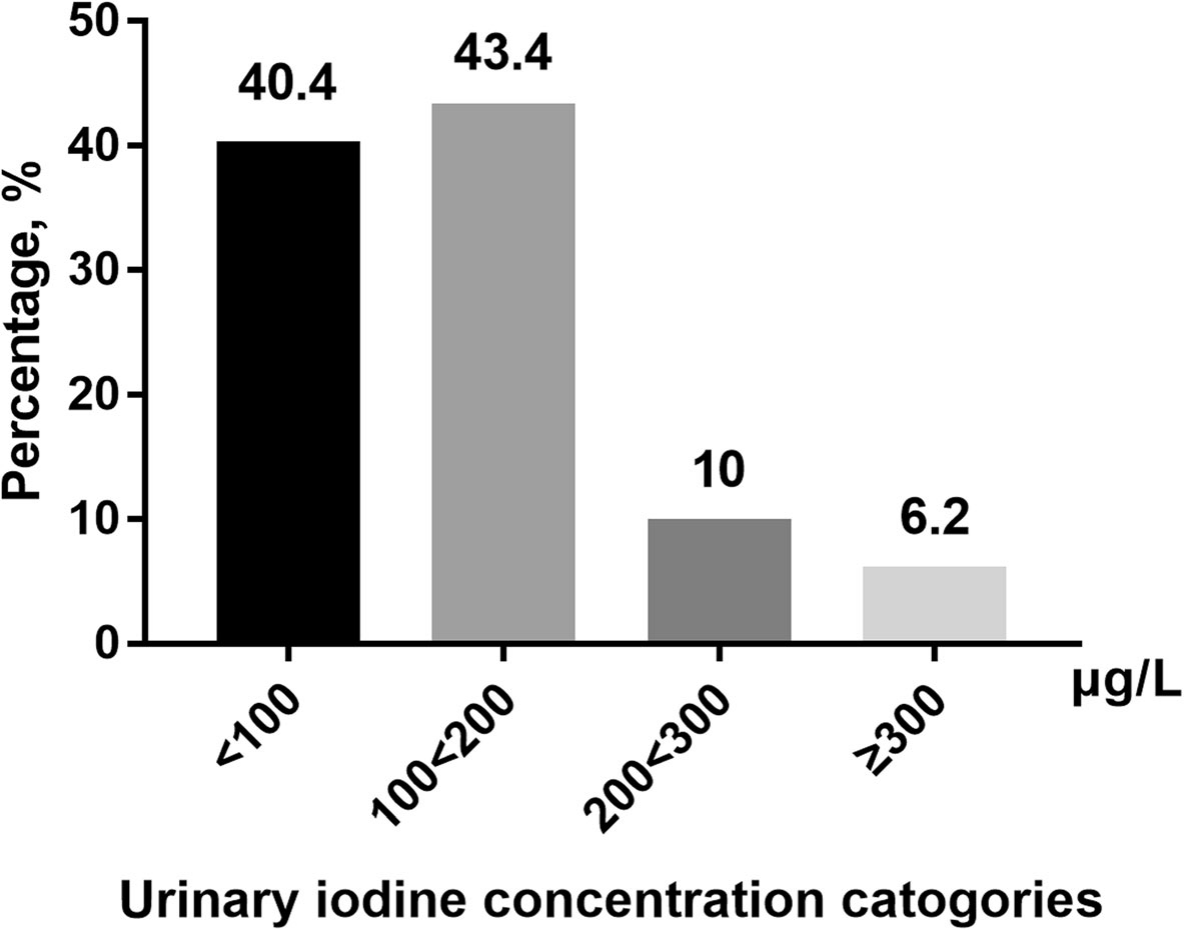
Distribution of UICs in the study population

The distribution of UICs in the study population is presented in Fig. 1. The median (25th–75th percentile) UIC of subjects with diabetes was 115.4 μg/L (78.9–170.8) in downtown Shanghai, which falls within the range of 100-199 μg/L that WHO/UNICEF/ICCIDD categorize as adequate. Urinary iodine measurements indicative of ID (UIC < 100 μg/L) were present in 40.4% of the study population. Meanwhile, 10.0 and 6.2% of the population showed more than adequate (UIC 200–299.9 μg/L) and excess iodine intake (UIC ≥ 300 μg/L), respectively.

**Fig. 2:**
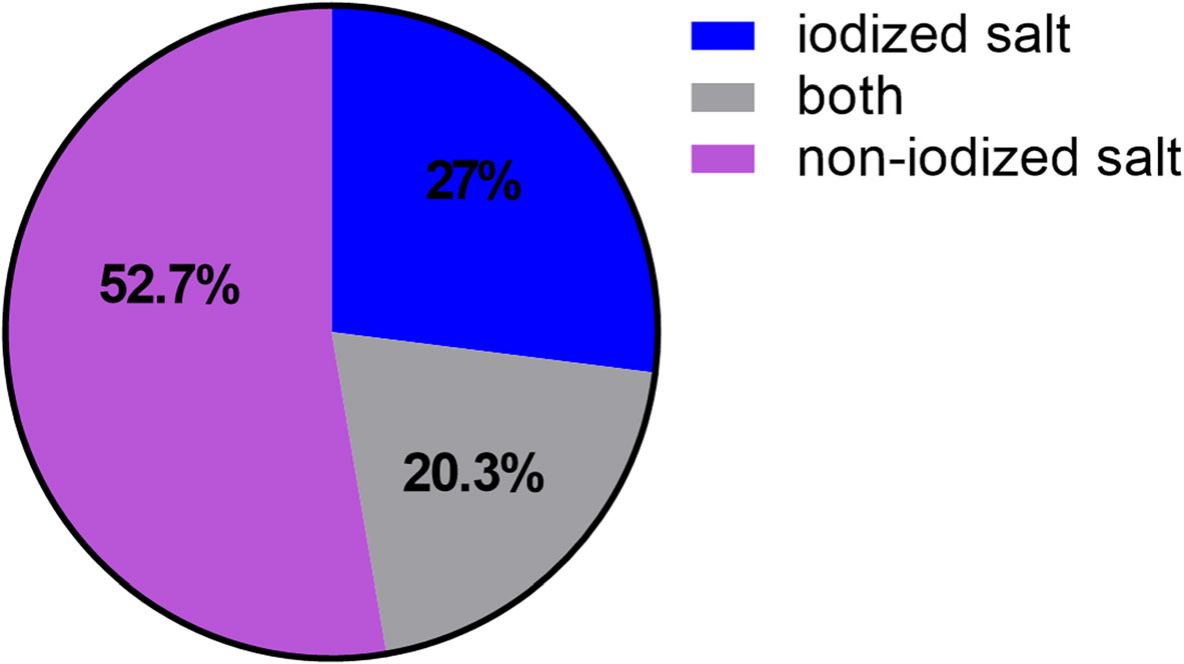
Distribution of type of salt consumed in the study population

The distribution of type of salt intake is presented in Fig. 2. As high as 52.7% of the study population consumed non-iodized salt, 27.0% consumed iodized salt, and 20.3% consumed both. Logistic regression analysis showed that compared to those who consumed iodized salt, subjects consumed non-iodized salt were more likely to be women (OR 1.27, 95%CI 1.10–1.47) and have a higher educational attainment (OR 1.29, 95%CI 1.12–1.50), but a comparable age (OR 0.99, 95%CI 0.98–1.01).

### Association of urinary iodine concentration with elevation of UACR, reduction of eGFR and DKD

The association of UIC with elevation of UACR, reduction of eGFR and DKD is shown in Table 2. Compared with those with adequate iodine nutrition, subjects with ID had an increased risk of elevation of UACR (OR 1.17, 95%CI 1.02–1.36), reduction of eGFR (OR 1.35, 95%CI 1.03–1.79) and DKD (OR 1.19, 95%CI 1.04–1.37). Adjustment for age, sex, education, current smokers, BMI, HbA1C, duration of diabetes, dyslipidemia, TSH and FT4 did not attenuate the association of ID with UACR and DKD. However, multivariable adjustment weakened the association between ID and reduction of eGFR further such that it was no longer significant. Meanwhile, subjects with more than adequate and excess iodine nutrition did not have an increased risk of elevation of UACR, reduction of eGFR and DKD after multivariable adjustment.

**Table 2:** Association of urinary iodine with elevation of UACR and reduction of eGFR

Urinary iodine	Adequate	Low	More than adequate	Excessive
**High UACR**				
Prevalence,%	24.4	27.5	24.6	24.2
Odds Ratio				
Model 1	1.0 (Ref.)	1.17 (1.02–1.36)*	1.01 (0.80–1.28)	0.99 (0.74–1.32)
Model 2	1.0 (Ref.)	1.17 (1.01–1.38)*	0.95 (0.73–1.23)	0.97 (0.70–1.34)
**Reduced eGFR**				
Prevalence, %	4.9	6.5	3.3	7.8
Odds Ratio				
Model 1	1.0 (Ref.)	1.35 (1.03–1.79)*	0.67 (0.38–1.16)	1.67 (1.03–2.70)*
Model 2	1.0 (Ref.)	1.17 (0.86–1.58)	0.67 (0.36–1.22)	1.48 (0.85–2.58)
**DKD**				
Prevalence, %	26.2	29.8	26.2	28.5
Odds Ratio				
Model 1	1.0 (Ref.)	1.19 (1.04–1.37)*	1.00 (0.79–1.26)	1.12 (0.85–1.48)
Model 2	1.0 (Ref.)	1.17 (1.01–1.37)*	0.93 (0.72–1.21)	1.13 (0.83–1.54)

Data are expressed as odds ratios (95%CI). Logistic regression analyses were used for the association of urinary iodine with elevation of UACR, reduction of eGFR and DKD. *P < 0.05

Model 1 was unadjusted

Model 2 was adjusted for age, sex, education, current smokers, BMI, HbA1C, duration of diabetes, dyslipidemia, TSH and FT4

High UACR was defined as UACR ≥30 mg/g, reduced eGFR as eGFR <60 ml/min/1.73 m^2^, and DKD as UACR > 30 mg/g or eGFR < 60 mL/min/1.73 m^2^

Urinary iodine concentrations: low, < 100 μg/L; adequate, 100 to < 200 μg/L; more than adequate, 200 to < 300 μg/L; excessive, ≥300 μg/L

### Association of urinary iodine concentration with DR

Table 3 presents the association of UIC with non-proliferative and proliferative DR. Compared with those with adequate iodine nutrition, the ORs of non-proliferative and proliferative DR in subjects with more than adequate iodine nutrition were 0.73 (95%CI 0.54–0.99) and 0.35 (95%CI 0.08–1.49), respectively. Multivariable adjustment weakened the association between more than adequate iodine nutrition and non-proliferative DR further such that the association was no longer significant. There was no significant association observed between DR and ID and excess iodine nutrition.

**Table 3:** Association of urinary iodine with DR

Urinary iodine	Adequate	Low	More than adequate	Excessive
**Non-proliferative DR**				
Prevalence, %	16.5	16.1	21.2	13.1
Odds Ratio				
Model 1	1.0 (Ref.)	1.03 (0.84–1.26)	0.73 (0.54–0.99)*	1.31 (0.83–2.05)
Model 2	1.0 (Ref.)	0.99 (0.80–1.23)	0.73 (0.53–1.00)	1.34 (0.83–2.15)
**Proliferative DR**				
Prevalence, %	0.4	0.5	1.0	0.5
Odds Ratio				
Model 1	1.0 (Ref.)	0.77 (0.23–2.53)	0.35 (0.08–1.49)	0.69 (0.08–5.98)
Model 2	1.0 (Ref.)	0.71 (0.22–2.38)	0.35 (0.08–1.51)	0.83 (0.09–7.26)

*P < 0.05

Data are expressed as odds ratios (95%CI). Logistic regression analyses were used for the association of urinary iodine with DR.

Model 1 was unadjusted

Model 2 was adjusted for age, sex, education, current smokers, BMI, HbA1C, duration of diabetes, dyslipidemia, TSH and FT4

Urinary iodine concentrations: low, < 100 μg/L; adequate, 100 to < 200 μg/L; more than adequate, 200 to < 300 μg/L; excessive, ≥300 μg/L

## Discussion

In this study among over 4500 community-dwelling Chinese adults with diabetes, we found that 52.7% of the subjects consumed non-iodized salt, and 40.4% had ID. Iodine deficiency was significantly associated with a higher prevalence of elevated UACR and DKD, independently of age, sex, education, current smokers, BMI, HbA1C, duration of diabetes, dyslipidemia, TSH and FT4. To the best of our knowledge, this is the first study to investigate the current status of iodized salt consumption and iodine nutrition status in a relatively large population with diabetes, and further investigate the association between UIC and diabetic microvascular complications.

China was once severely affected by IDD, and hence, a mandatory Universal Salt Iodization (USI) program was introduced in 1996, and was successful in eliminating IDD. However, since the prevalence of thyroid diseases has markedly increased in recent years, some concerns about the USI have circulated, especially among coastal residents in urban areas [21]. In the present analysis, the median UIC of residents with diabetes in downtown Shanghai has fallen to marginal levels of iodine sufficiency (115.4 μg/L), and more surprisingly, more than half (52.7%) of the subjects consumed non-iodized salt and 40.4% were iodine deficient. Compared with a study conducted by the Shanghai Municipal Center for Disease Control and Prevention (CDC) in 2009, 95.3% of participants used iodized salt and 28.6% were iodine deficient at that time [22]. During 2011–2012, Zhongyan Shan and her colleagues performed a cross-sectional study in eastern and central China and reported that the median UIC was 197 μg/L in school-aged children and 205 μg/L in the total cohort population [23]. Our previous study conducted on the general population found that 46.4% consumed non-iodized salt in the urban area of Shanghai in 2016 [24].

The present study indicates that an increasing number of urban residents in downtown Shanghai prefer to use non-iodized salt in recent years and suffer from ID. Women and those with a higher educational level were more tended to consume non-iodized salt. Why? Changes in the reported spectrum and growing incidence of thyroid disorders have been linked to the increased iodine intake resulting from USI in the local media and international medical literature [25]. Those with high educational attainment were more likely to be confused by these information and worry about their thyroid health, and call for liberalizing provincial control of sales of non-iodized salt. Formerly needed a prescription, the sale of non-iodized salt has now been unofficially allowed by some coastal city authorities [26]. In addition, T2DM is associated with an increased risk of multiple thyroid disorders such as thyroid nodule [27], thyroid cancer [28], and autoimmune thyroid diseases [29]. Since the prevalence of *thyroid* abnormalities were found *to* be much higher *in* females than males [30], it is reasonable to deduce that these women would have a higher tendency towards consuming non-iodized salt after diagnosis of thyroid abnormalities.

Considering that Shanghai is a coastal city, local residents believe that they should never suffer from IDD with high iodine-enriched aquatic products consumption. However, it may not be true to rely on consumption of seafood alone to provide sufficient iodine. Actually, the environmental levels of iodine in Shanghai are deficient (< 10 μg/L) [23]. Furthermore, based on the research initiated by Shanghai Municipal CDC, iodized salt contributed 63.5% of the total dietary iodine in Shanghai [22]. Aquatic products, which residents thought to be rich in iodine, accounted for only 5.03% of the total dietary iodine [22].Thus, iodized salt is still the main source for iodine supplementation in coastal populations.

DKD was commonly defined by ADA as “UACR≥30mg/g or eGFR<60 ml/min per 1.73 m^2^” [31]. We found that ID was associated with elevated UACR and DKD. The mechanism underlying the association of ID with DKD is not yet fully understood. Deficiency of iodine could reduce thyroid hormone production and elevate TSH. It has been reported that lower free triiodothyronine and elevated TSH have significant association with risk for albuminuria in T2DM [32, 33]. Moreover, inadequate iodine intake is significantly correlated with an increase in oxidative stress [34]. Recent evidence has shown that inflammatory cytokines such as tumor necrosis factor-alpha and interleukin-1 play a pivotal role in the pathogenesis of DKD [35]. Therefore, we suppose the possible mechanism may be via inflammatory response.

In view of the ever-growing prevalence of T2DM and DKD all over the world, successive intervention in this large population can have important impact on public health. ID, unlike most micronutrient deficiencies, is not restricted to people in developing countries with poor diets. Since salt iodization is simple, effective and inexpensive, the best strategy to control ID is addition of iodine into salt in nearly all countries [36]. Monitoring iodine situation of people with diabetes is of critical significance and education programs to diabetes, especially women with high academic background, may also include information of adequate iodine intake in our clinical practice. Our study shed light on the possible beneficial effect of iodine supplementation in reducing albuminuria in T2DM, which warrants further investigation in well-designed randomized controlled trial.

Our study benefited from its well-defined community-based participants with a relatively large sample size. Second, regarding the novelty, our study is the first to provide iodine status, and linked iodine insufficiency to an increased risk of albuminuria in people with diabetes. Third, we used ICP-MS to detect UIC in the present analysis, which was considered as the gold-standard method [37]. There were also some limitations we should acknowledge. First, no causal relationship could be determined due to the cross-sectional design of the study, and thus our findings need to be validated by longitudinal prospective studies. Second, although UIC is recommended by the WHO/ICCIDD/UNICEF for evaluation of iodine status at the population level and widely used in large-scale epidemiological studies [38, 39], the single spot urine measurement may not accurately assess long-term iodine status at the individual level. Actually, inter- and intra-individual variability exists in UIC [40]. However, the application of a large sample size (from 100 to 500 subjects per sub-group) may counteract the bias related to the use of only one casual urine sample [41]. Future follow-up studies collecting 24-h urine specimens twice are needed to replicate the present results.

## Conclusion

A large proportion of diabetic patients in downtown Shanghai consumed non-iodized salt and had ID. ID may increase the risk of DKD independent of thyroid function in diabetic patients. Maintaining USI at an appropriate level is indispensable for diabetic patients. Cohort and intervention studies as well as basic research exploring the effect and mechanism of iodine supplementation on renal function are warranted.

## Data Availability

The datasets during and/or analyzed during the current study are available from the corresponding author on reasonable request.

## Abbreviations

T2DM: Type 2 Diabetes Mellitus
DKD: Diabetic kidney disease
DR: Diabetic retinopathy
ID: Iodine deficiency
UIC: Urinary iodine concentration
TSH: Thyroid-stimulating hormone
BMI: Body mass index
OR: Odds ratio
CI: Confidence interval
UACR: Urinary albumin-to-creatinine ratio
eGFR: Estimated glomerular filtration rate
